# Alcoholic Hepatitis and Intestinal Barrier Breakdown: A Theoretical Reappraisal Based on Pentoxifylline's Action

**DOI:** 10.4021/gr2009.05.1288

**Published:** 2009-05-20

**Authors:** Stelios F. Assimakopoulos, Konstantinos C. Thomopoulos, Chrisoula Labropoulou-Karatza

**Affiliations:** a Department of Internal Medicine, University Hospital of Patras, Patras 26504, Greece; b Division of Gastroenterology, Department of Internal Medicine, University Hospital of Patras, Patras 26504, Greece

Alcoholic hepatitis is one of the most serious forms of alcoholic liver injury, associated with significant early mortality, as inpatient mortality can attain 50-75% in the most severe forms [1, 2]. The treatment of alcoholic hepatitis remains one of the main challenges to clinicians involved in the management of severe alcoholic liver disease and represents one of the most debated topics in medicine and a field of continued research.

While the histology of alcoholic hepatitis is well characterized, its pathogenesis has not been clearly explained and is an era of active research. A number of hepatocellular and inflammatory processes including the potential involvement of innate and adaptive immunologic responses, oxidative stress and impaired hepatic regeneration are under investigation [3, 4]. On this basis, diverse treatment options (glucocorticoids, pentoxifylline, infliximab, s-adenosyl-methionine and colchicine) have been tried in alcoholic hepatitis with a common goal of blocking the release of chemokines and cytokines by inflammatory cells in response to alcohol-associated oxidant stress and endotoxemia [5].

Current guidelines recommend the use of glucocorticosteroids for patients with severe alcoholic hepatitis as defined by the Maddrey score (discriminant-function ≥32) [5, 6]. However, their use is limited in cases of gastrointestinal bleeding or active infection. Amongst other available treatment options that have already been tested, only pentoxifylline (PTX) may be considered as an alternative to corticosteroids for severe acute alcoholic hepatitis based on the favourable results of studies comparing PTX with placebo [7, 8]. Although primary treatment of severe alcoholic hepatitis with PTX is not currently recommended due to lack of evidence of its efficacy as compared to the standard treatment with corticosteroids, a very recent study by De BK et al [9] compared the two treatments head to head in a randomized controlled manner and demonstrated the superiority of PTX over corticosteroids, in terms of patients' survival and risk-benefit profile.

Up to now, the use of PTX in severe alcoholic hepatitis has been supported by three clinical studies. The first one was conducted by McHutchison et al in 1991, in patients with severe alcoholic hepatitis (defined as DF score ≥ 32) and showed that PTX reduced the development of hepatorenal syndrome, and as a consequence mortality, in comparison to patients who received placebo [7]. These findings were confirmed in 2000 by Akriviadis et al [8] in a double-blind placebo-controlled trial. In the latter study, twenty-four percent of PTX treated patients and 46.1% of control patients died during hospitalization. The survival benefit of PTX appeared to be related to a significant reduction of hepatorenal syndrome. Among patients who died, hepatorenal syndrome had developed in 50% of PTX patients and in 91.7% of placebo patients. The third and very recent study was conducted by De BK and colleagues [9], in a randomized double-blind controlled study, the efficacy of PTX was evaluated over the standard treatment of prednisolone in the management of severe alcoholic hepatitis (DF ≥ 32). This study showed a significantly reduced mortality among patients in the PTX group (14.71%) compared to those receiving prednisolone (35.29%, *P* = 0.04), accompanied by an advantageous safety profile. Reduction of mortality in the PTX group was again related to a significant reduction of hepatorenal syndrome. Among patients who died, hepatorenal syndrome had developed in 50% of prednisolone treated patients and in none of PTX treated patients.

The above stated studies have demonstrated that survival advantage in PTX treated patients with severe alcoholic hepatitis is clearly related to prevention of hepatorenal syndrome [7-9]. The mechanism of this beneficial effect is not evident by available data and only speculations could be made on this matter. PTX, a non-specific phosphodiesterase inhibitor, is a suppressor of tumor necrosis factor alpha (TNF-α), prevents leukocyte adherence to vascular endothelium, down-regulates the expression of intercellular adhesion molecule-1 in monocytes and improves the rheological properties of blood [10]. The rationale of its use in alcoholic hepatitis is based on its property to inhibit the synthesis of TNF-α, which is considered as a pivotal mediator of liver injury in alcoholic hepatitis [4, 8]. However, prevention of hepatorenal syndrome and survival advantage in patients with severe alcoholic hepatitis treated with PTX was not associated with decreased circulating TNF-α levels or with improved liver function [8]. These findings point towards an alternative explanation of PTX's positive effect on renal function in alcoholic hepatitis, but before any assumption is made on this issue it is essential to review the pathophysiology of hepatorenal syndrome with a special focus on alcoholic hepatitis-associated hepatorenal syndrome.

Hepatorenal syndrome is defined as a specific type of functional renal failure complicating advanced liver disease (e.g. decompensated cirrhosis, alcoholic hepatitis, or acute liver failure). The pathogenesis of this syndrome is the result of an extreme underfilling of the arterial circulation secondary to an arterial vasodilation located in the splachnic circulation. This underfilling triggers a compensatory response with activation of the sympathetic nervous system and release of humoral and renal vasoactive mediators leading to intense renal vasoconstriction, especially to the renal cortex, and decreased glomerular filtration rate [11, 12]. In the induction of systemic vasodilatation, which is the primary event in the development of hepatorenal syndrome, systemic endotoxemia is considered to play a pivotal role [12]. In patients with alcoholic hepatitis, the existence and significant role of systemic endotoxemia on liver injury has been shown repeatedly and its pathogenesis has been well described [13, 14]. Alcoholic hepatitis induces a significant increase of intestinal permeability to macromolecules thus permitting the escape of endotoxin from the gut into portal circulation and consequently a depressed elimination of endotoxin by Kupffer cells induces spillover endotoxemia [3, 15]. Processing of endotoxin by Kupffer cells and mainly by extrahepatic macrophages induces cytokinemia [3, 15]. Systemic endotoxemia per se and endotoxin-induced cytokinemia further increase intestinal permeability through modulation of intestinal tight junctions and the vicious cycle of endotoxemia is maintained [16]. In view of this pathophysiological sequence of events, which potential mechanism(s) of action could explain the positive impact of PTX on prevention of hepatorenal syndrome and survival advantage in alcoholic hepatitis?

One potential mechanism could be PTX's positive effect on splachnic and renal microcirculation and this explanation, based on the hemorheological properties of PTX, was proposed in the initial study by Akriviadis et al [8]. However, given the central role of gut-derived endotoxemia in the pathophysiology of hepatorenal syndrome and considering also the significant prevention of hepatorenal syndrome in severe alcoholic hepatitis by PTX administration, as the study by DE BK et al [9] showed total prevention of hepatorenal syndrome, we are tempting to speculate that PTX should inevitably act, at least partly, through reduction of systemic endotoxemia. In other words, PTX might act through maintenance of an effective gut barrier function which prevents endotoxin translocation from gut lumen into portal circulation. Up to now, there is considerable evidence to support this theory. Experimental studies have shown that PTX and its chemically relevant molecule lisofylline effectively prevent intestinal barrier breakdown after various injurious insults like burns, sepsis, hemorrhagic shock or ischemia-reperfusion [17-21]. This protective action on gut barrier function is the result of its regulatory effect on the tight junctions’ structure and function, thus leading to preservation of paracellular permeability. Specifically, PTX preserves the expression and cellular distribution of the key tight junction associated molecules occludin and ZO-1, possibly through attenuation of activation of the important tight junction modulator myosin light chain kinase (MLCK) [17-19]. Maintenance of gut barrier function might also be an important part of the positive effect of glucocorticoids in alcoholic hepatitis, beyond their widely accepted mechanisms of action, which is blocking of cytokine release and their potent anti-inflammatory action. Previous studies have demonstrated that glucocorticoids can enhance the intestinal tight junction barrier through inhibition of the TNF-α-induced increase in myosin light chain kinase (MLCK) protein expression, a key process mediating the TNF-α increase in intestinal tight junction (TJ) permeability [22]. However, one could ask why the potential prevention of gut barrier function and amelioration of systemic endotoxemia by PTX administration was not accompanied by a concomitant reduction of TNF-α levels and liver injury. One potential answer might be that endotoxin is not the only promoter of TNF-α production in alcoholic hepatitis. Previous studies have shown that there is no correlation between plasma endotoxin and TNF-α levels, whilst lipid peroxidation products and oxygen free radicals are important promoters of TNF-α production as well [23].

In conclusion, we believe that asking “why” in every step of the diagnostic and therapeutic process and attempting to find convincing answers should be a continuous task of all clinicians. Our question on the potential mechanism of action of PTX in severe alcoholic hepatitis, lead us, through a theoretical approach, to reconsider the importance of gut barrier function in the outcome of these patients. Future research may confirm or refute our speculations on PTX's mechanism of action. However, pending convincing answers on this issue, we think that we should augment our focus on protecting our patients' intestinal barrier during hospitalization because of severe alcoholic hepatitis, beyond applying the specific pharmacological strategies of glucocoricoids or PTX. This could be achieved by simple or more specific measures, such as adequate fluid replacement to prevent visceral-microcirculatory disturbances, enteral nutrition to improve microcirculation, prevent mucosal atrophy and provide important nutrients for enterocytes, lactulose administration to reduce intraluminal endotoxin load or by selective gut decontamination. In our opinion, understanding in a global way the alterations induced over time in the epithelial cell lining of the intestine in alcoholic hepatitis and discovering selective modulators of intestinal tight junctions or other structures critically involved, in order to control intestinal hyperpermeability, is an emerging and attractive field of research.
